# Intercontinental validation of a clinical prediction model for predicting 90-day and 2-year mortality in an Israeli cohort of 2033 patients with a femoral neck fracture aged 65 or above

**DOI:** 10.1007/s00068-023-02237-5

**Published:** 2023-02-09

**Authors:** Jacobien H. F. Oosterhoff, Aditya V. Karhade, Olivier Q. Groot, Joseph H. Schwab, Marilyn Heng, Eyal Klang, Dan Prat

**Affiliations:** 1 Department of Orthopaedic Surgery, Amsterdam Movement Sciences, Amsterdam University Medical Centers, University of Amsterdam, Meibergdreef 9, 1105AZ Amsterdam, the Netherlands; 2 Department of Orthopaedic Surgery, Massachusetts General Hospital, Harvard Medical School, Boston, MA USA; 3 Department of Orthopaedic Surgery, University of Miami Miller School of Medicine, Miami, FL USA; 4 Orthopaedic Trauma Service, Jackson Memorial Ryder Trauma Center, Miami, FL USA; 5 Sami Sagol AI Hub, ARC, Sheba Medical Center, Ramat Gan, Israel; 6 Department of Orthopaedic Surgery, Sheba Medical Center, Ramat Gan, Israel; 7 Department Engineering Systems and Services, Faculty Technology Policy and Management, Delft University of Technology, Delft, The Netherlands

**Keywords:** Hip fracture, Femoral neck fracture, Geriatric trauma, Prediction model, Mortality, Machine learning

## Abstract

**Purpose:**

Mortality prediction in elderly femoral neck fracture patients is valuable in treatment decision-making. A previously developed and internally validated clinical prediction model shows promise in identifying patients at risk of 90-day and 2-year mortality. Validation in an independent cohort is required to assess the generalizability; especially in geographically distinct regions. Therefore we questioned, is the SORG Orthopaedic Research Group (SORG) femoral neck fracture mortality algorithm externally valid in an Israeli cohort to predict 90-day and 2-year mortality?

**Methods:**

We previously developed a prediction model in 2022 for estimating the risk of mortality in femoral neck fracture patients using a multicenter institutional cohort of 2,478 patients from the USA. The model included the following input variables that are available on clinical admission: age, male gender, creatinine level, absolute neutrophil, hemoglobin level, international normalized ratio (INR), congestive heart failure (CHF), displaced fracture, hemiplegia, chronic obstructive pulmonary disease (COPD), history of cerebrovascular accident (CVA) and beta-blocker use. To assess the generalizability, we used an intercontinental institutional cohort from the Sheba Medical Center in Israel (level I trauma center), queried between June 2008 and February 2022. Generalizability of the model was assessed using discrimination, calibration, Brier score, and decision curve analysis.

**Results:**

The validation cohort included 2,033 patients, aged 65 years or above, that underwent femoral neck fracture surgery. Most patients were female 64.8% (*n* = 1317), the median age was 81 years (interquartile range = 75–86), and 80.4% (*n* = 1635) patients sustained a displaced fracture (Garden III/IV). The 90-day mortality was 9.4% (*n* = 190) and 2-year mortality was 30.0% (*n* = 610). Despite numerous baseline differences, the model performed acceptably to the validation cohort on discrimination (c-statistic 0.67 for 90-day, 0.67 for 2-year), calibration, Brier score, and decision curve analysis.

**Conclusions:**

The previously developed SORG femoral neck fracture mortality algorithm demonstrated good performance in an independent intercontinental population. Current iteration should not be relied on for patient care, though suggesting potential utility in assessing patients at low risk for 90-day or 2-year mortality. Further studies should evaluate this tool in a prospective setting and evaluate its feasibility and efficacy in clinical practice. The algorithm can be freely accessed: https://sorg-apps.shinyapps.io/hipfracturemortality/.

**Level of evidence:**

Level III, Prognostic study.

**Supplementary Information:**

The online version contains supplementary material available at 10.1007/s00068-023-02237-5.

## Introduction

The number of hip fractures continues to rise and is predicted to have an incidence of 6 million cases each year worldwide in 2050 [1]. Numerous patient and injury characteristics are associated with a high mortality rate after hip fracture, with incidences up to 35% in the first year after surgery [2–4]. Mortality prediction and personalized risk management based on prognosis are essential to guide clinical decision-making and effective healthcare services [5, 6]. Considering rapid population aging, researchers are aiming at extending life duration, while, at the same time maximizing the quality of life, and minimizing the overall associated healthcare costs [7]. The development of models for the prediction of risk of death in trauma, in the critically ill and in intensive care unit patients, are common examples of use for such models [8–10]. Numerous predictors increasing the risk of mortality after hip fracture surgery have been identified by prospective, retrospective, and meta-analyses studies including patient and injury characteristics [11–15].

Recently, the clinical prediction model SORG Orthopaedic Research Group (SORG, previously Skeletal Oncology Research Group) using machine learning algorithms (MLA) was developed showing promise in estimating the risk of 90-day and 2-year mortality in 2478 femoral neck fracture patients aged 65 years or above in a multicenter institutional cohort from the USA [16]. The SORG-MLA is available in an open access web application: https://sorg-apps.shinyapps.io/hipfracturemortality/. Many promising clinical prediction models exist to predict mortality in hip fracture patients, but the vast majority of them are awaiting external validation [17]. External validation is required to assess the generalizability of the clinical prediction model in a geographically different patient population [18].

Therefore, in this study, we asked: Is the SORG femoral neck fracture mortality algorithm externally valid in an Israeli cohort of 2033 patients to predict 90-day and 2-year mortality?

## Materials and methods

### Data source

Patients were included when older than 65 years of age who underwent operative fixation of a femoral neck fracture. Patients were excluded when sustaining a pathological hip fracture or sustaining septic shock on admission. The primary outcome of interest was 90-day and 2-year mortality due to any cause following femoral neck fracture surgery.

The developmental cohort originated from the Massachusetts General Brigham hospitals. In total, 2478 patients were included with 90-day mortality proportion of 9.1% (225 of 2478) and 2-year mortality proportion of 23.5% (582 of 2478) [16]. The models included the following input variables that are available on clinical admission: age, male gender, creatinine level, absolute neutrophil, hemoglobin level, international normalized ratio (INR), congestive heart failure (CHF), displaced fracture, hemiplegia, chronic obstructive pulmonary disease (COPD), history of cerebrovascular accident (CVA) and beta-blocker use. The stochastic gradient boosting algorithm had the best performance for 90-day mortality prediction, with good discrimination (*c*-statistic = 0.74), calibration (intercept = − 0.05, slope = 1.11) and Brier score (0.078). The elastic-net penalized logistic regression algorithm had the best performance for 2-year mortality prediction, with good discrimination (*c*-statistic = 0.70), calibration (intercept = − 0.03, slope = 0.89) and Brier score (0.16). Further details of the original clinical prediction model can be found in the developmental study [16].

The validation cohort originated from the Sheba Medical Center in Israel (level I trauma center) and was queried from June 1st, 2008 to February 1st, 2022. Patients older than 65 years of age were identified who underwent operative treatment for a femoral neck fracture, OTA type 31-B (as classified by the Orthopaedic Trauma Association (OTA) [19]). Patients were excluded if presented with a pathological fracture.

The same outcome and variable definitions were used as the developmental cohort. The authors of the developmental study were not present during data extraction.

### Participants’ baseline characteristics

We included 2033 patients that were operatively treated following a femoral neck fracture, with 90-day mortality proportion of 9.4% (190 of 2033 patients) and 2-year mortality proportion of 30.0% (610 of 2033 patients). Of the included patients, 64.8% (1,317 of 2033 patients) were female, and the median age was 81 years (interquartile range [IQR] = 75–86) (Table 1). A majority of 80.4% (1635 of 2033 patients) sustained a displaced femoral neck fracture (Garden III/IV).

**Table 1:** Baseline characteristics of the developmental and validation cohorts

Variable	Developmental cohort (*n* = 2478)	Validation cohort (*n* = 2,033)	*p* value
Age	83 (76–88)	81 (75–86)	< .001
Female gender	69.5 (1723)	64.8 (1317)	< .001
Displaced fracture (Garden III/IV)	71.2 (1765)	80.4 (1635)	< .001
Comorbidities			
History of cerebrovascular accident	17.8 (442)	13.3 (270)	< .001
Congestive heart failure	29.0 (718)	6.8 (139)	< .001
Hemiplegia	2.4 (60)	1.1 (22)	< .001
Dementia	12.5 (309)	9.9 (201)	< .01
Chronic obstructive pulmonary disease	26.6 (658)	3.4 (69)	< .001
Beta-blocker use	51.9 (1287)	48.2 (980)	< .05
Laboratory values			
Hemoglobin	12.1 (11.0–17.8)	12.3 (11.3–13.4)	< .001
Creatinine	0.93 (0.74–1.21)	0.91 (0.75–1.19)	0.29
Absolute lymphocyte	1.14 (0.82–1.55)	1.19 (0.85–1.61)	0.06
Absolute neutrophil	7.77 (5.62–8.27)	7.53 (5.64–10.1)	0.22
INR	1.1 (1.0–1.2)	1.0 (0.95–1.09)	< .001
Mortality			
90-day	9.1 (225)	9.4 (190)	0.76
2-year	23.5 (582)	30.0 (610)	< .001

Data presented as % (*n*) for dichotomous/categorical variables and as median (interquartile range) for continuous variables

*ASA* American Society of Anesthesiologists

### Missing data

Pre-processing of the validation cohort was carried out by imputing missing values using the missForest methodology [20], as previously applied in the development paper [21–25]. We imputed missing values for the following laboratory variables: hemoglobin (5.9% [119 of 2,033]), absolute lymphocyte (6.0%, [122 of 2,033]), absolute neutrophil (6.0%, [122 of 2,033]), creatinine (6.4%, [129 of 2,033]) and INR (13.2%, [269 of 2,033]). No missing data for 90-day and 2-year mortality were observed.

### Model performance

Model performance was evaluated according to a proposed framework for evaluation of a clinical prediction model [26] that includes: (1) discrimination with the c-statistic, (2) calibration with calibration slope and intercept (in-line with the method by Cox [27]) and (3) the overall performance with the Brier score.

The c-statistic (area under the curve of a receiver operating characteristic curve) is a score ranging from 0.5 to 1.0 with 1.0 indicating the highest discrimination score and 0.5 indicating the lowest. The higher the discrimination score, the better the model’s ability to distinguish patients who got the outcome from those who did not [28].

A calibration plot plots the estimated versus the observed probabilities for the primary outcome. A perfect calibration plot has an intercept of 0 (< 0 reflects overestimation, > 0 reflects underestimating the probability of the outcome) and a slope of 1 (the model is performing similarly in training and test sets) [26, 29]. In a small dataset, the slope is often < 1 reflecting model overfitting; probabilities are too extreme (low probability too low, high probability too high) [28].

The Brier score calculates a composite of discrimination and calibration, with 0 indicating perfect prediction and a Brier score of 1 the poorest prediction. The Brier score reflects the model to measure the accuracy of a predicted probability, compared to the actual outcome. The null model Brier score is a reflection of the average actual probability [26].

### Decision curve analysis

In addition, a decision curve analysis was undertaken and visualized to investigate the net benefit (weighted average of true positives and false positives) of the conducted algorithms over the range of risk thresholds for clinical decision-making [30]. The net benefit is a weighted average of true positives and false positives, formula = sensitivity × prevalence – (1−specificity) × (1 – prevalence) × (odds at the threshold probability). With threshold probability, we refer to the probability that an algorithm ranks a ‘positive’ outcome over a ‘negative’ outcome. In this study, a ‘positive outcome’ is someone at high risk of mortality in 90 days or 2 years. If the threshold is set at 0.5, then patients with a probability > 0.5 are classified as ‘positive’, and < 0.5 are classified as ‘negative’. If the threshold is set at 0.8, then patients with a probability > 0.8 are classified as ‘positive’, and < 0.8 are classified as ‘negative’. The decision curve of the model is compared to decision curves of treating everyone as being at risk for shorter- or longer-term mortality (depending on the endpoint) and treating no one as being at risk.

### Statistical analysis

Variables of the baseline characteristics were presented with frequencies and percentages for dichotomous and categorical variables, and median with IQR for continuous variables. Baseline characteristics of the developmental and validation cohort were compared using bivariate analysis, where a *p*-value of < 0.05 was considered significant.

### Guidelines

This study followed the Transparent Reporting of Multivariable Prediction Models for Individual Prognosis or Diagnosis Guideline (TRIPOD-Statement) (Supplemental Table 1) [31].

### Software

Data pre-processing and analysis were performed using R Version 4.0 (“R: A Language and Environment for Statistical Computing" The R Foundation, Vienna, Austria 2013) and R-studio Version 1.2.1335 (R-Studio, Boston, MA, USA).

## Results

### Participants

Baseline characteristics in the validation cohort (Israel) differed from those in the original developmental cohort (USA) [16] in several regards (Table 1). The Israeli cohort had a slightly younger age, a higher percentage of male gender, a lower proportion of patients using a beta-blocker, and fewer comorbidities (all *p* < 0.05). The 90-day mortality rate was comparable in the validation cohort compared to the developmental cohort (9.4% *versus* 9.1%, *p* = 0.76), but the 2-year mortality rate was higher in the validation cohort (Israeli) than in the developmental cohort (30.0% *versus* 23.5%, *p* < 0.001).

### Is the SORG femoral neck fracture mortality algorithm externally valid in an Israeli cohort to predict 90-day and 2-year mortality?

**Fig. 1:**
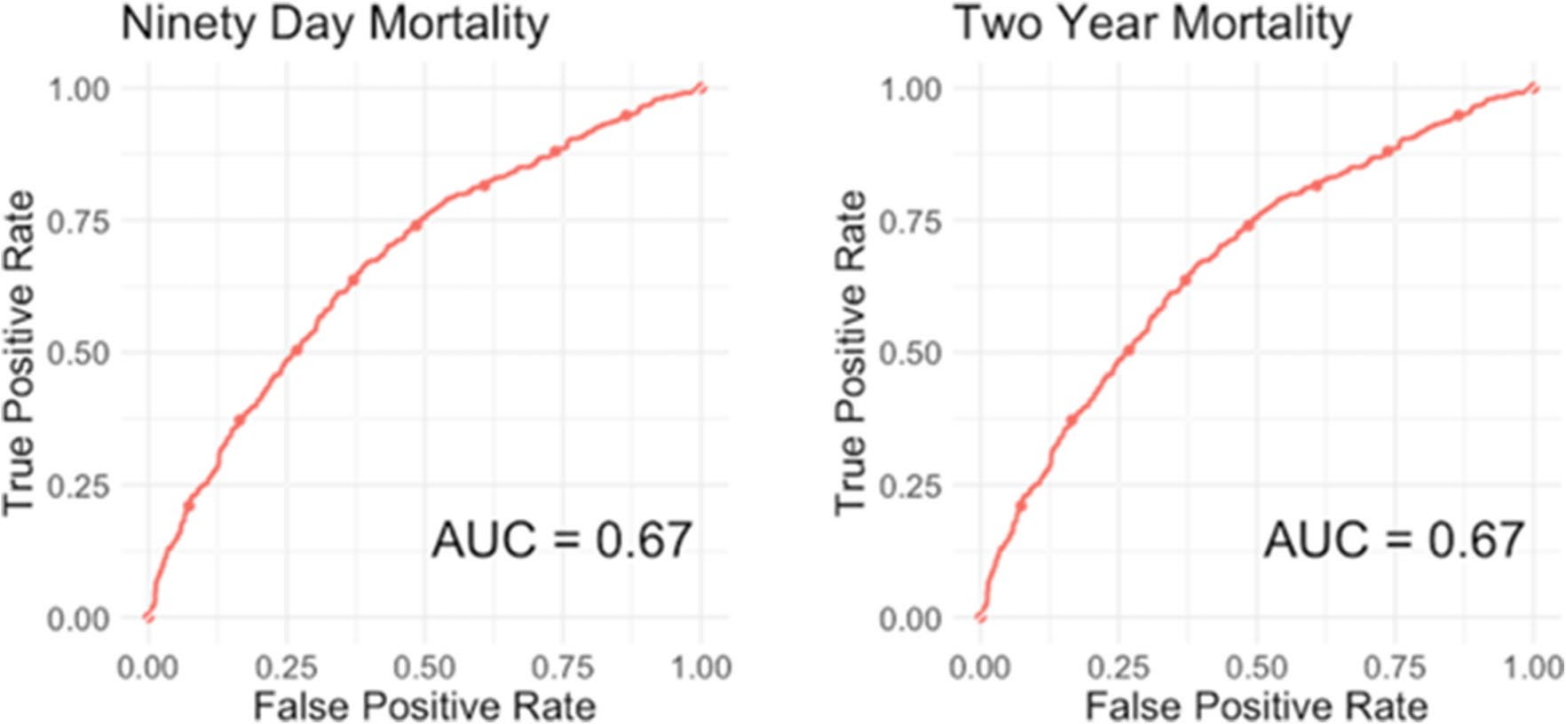
Receiver operating curves for SORG femoral neck fracture mortality algorithm on external validation, *n* = 2,033

**Fig. 2:**
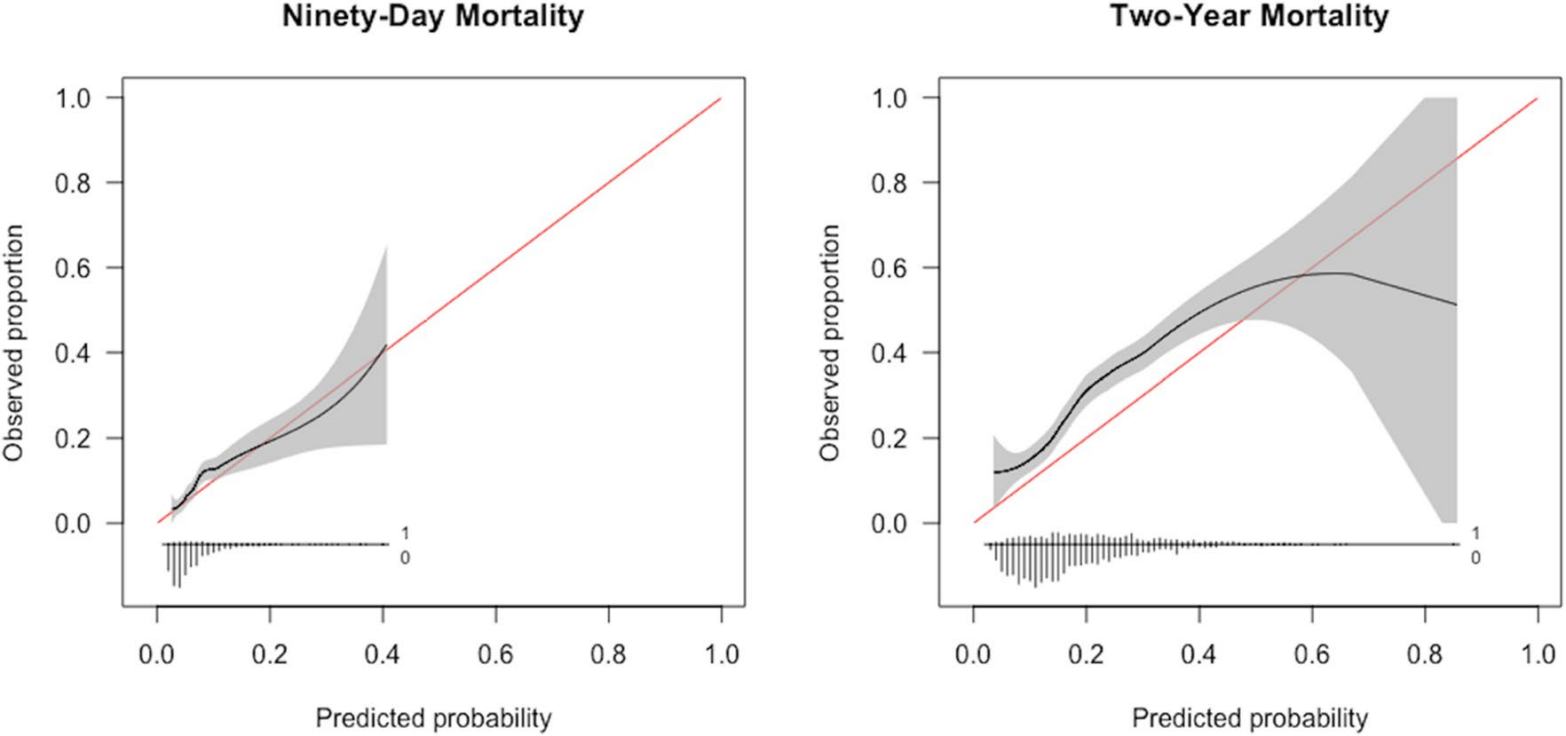
Calibration plots for SORG femoral neck fracture mortality algorithm on external validation, *n* = 2033

**Fig. 3:**
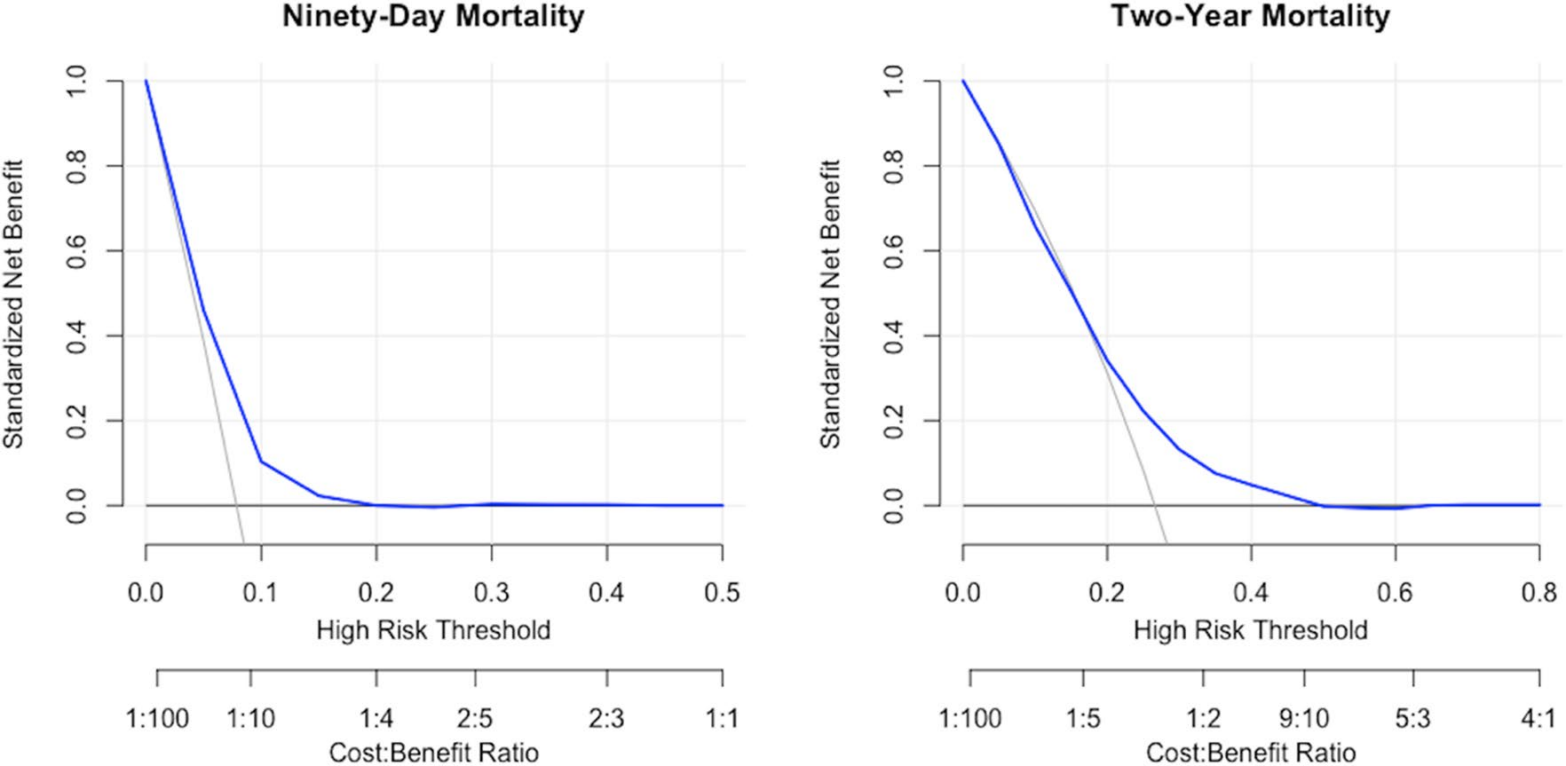
Decision curve analysis for SORG femoral neck fracture mortality algorithm on external validation, *n* = 2033

The SORG femoral neck fracture mortality algorithm achieved acceptable discrimination in predicting 90-day and 2-year mortality femoral neck fracture patients aged 65 years or above in the Sheba Medical Center cohort. For 90-day mortality prediction, the c-statistic was 0.67 (95% confidence interval [CI] 0.62 to 0.71) (Table 2), (Fig. 1). The calibration plot of the algorithm in the validation cohort showed calibration metrics with an intercept of 0.18 (95% CI 0.02 to 0.35) and a calibration slope of 0.92 (95% CI 0.67 to 1.17) (Fig. 2). The Brier score was lower than the respective null model Brier score (0.071 versus 0.073) indicating good overall performance of the SORG femoral neck fracture mortality algorithm. In the decision curve analysis, the SORG femoral neck fracture mortality algorithm has shown to provide a positive net benefit compared with a strategy of treating all patients or none as being at risk for 90-day mortality (Fig. 3). The model especially performs well in predicting patients at risk of 90-day mortality up to 40% risk.

**Table 2:** Model performance assessment on external validation in the Sheba Medical Center cohort (95% CI), *n* = 2,033

Reference: Model performance metrics in the development and internal validation cohort				
Metric	90-day mortality	Reference	2-year mortality	Reference
c-statistic ^a^	0.67 (0.62, 0.71)	*0.74 (0.67, 0.80)*	0.67 (0.65, 0.70)	*0.70 (0.63, 0.75)*
Intercept ^b^	0.18 (0.02, 0.35)	*− 0.05 (− 0.37, 0.26)*	0.50 (0.40, 0.61)	*− 0.03 (− 0.27, 0.19)*
Slope ^b^	0.92 (0.67, 1.17)	*1.11 (0.73, 1.51)*	0.90 (0.74, 1.04)	*0.89 (0.62, 1.19)*
Brier ^c^	0.071 (0.062, 0.081)	*0.078 (0.061, 0.098)*	0.19 (0.18, 0.20)	*0.16 (0.15, 0.18)*

Null-model Brier score in the Israeli cohort: 90-day—0.073, 2-year—0.20

^a^A *c*-statistic of 0.5 indicates random guess and 1.0 indicates perfect discriminatory ability; a *c*-index of 0.6 to 0.7 is typically considered acceptable discriminatory ability

^b^Calibration plots the predicted versus the observed probabilities; a perfect calibration plot has an intercept of 0 (< 0 reflects overestimation and > 0 reflects underestimating the probability of the outcome) and a slope of 1 (model is performing similarly in training and test sets); if the slope is < 1 (often in small datasets), this reflects model overfitting; probabilities are too extreme (low probability too low; high probability too high)

^c^The Brier score of the prediction model should be compared with that of the null model; the null-model Brier score is a score calculated from the probability of delirium in the dataset and used to benchmark the algorithm’s Brier score; a lower Brier score of the prediction model indicates good overall model performance

For 2-year mortality prediction, the c-statistic was 0.67 (95% CI 0.65 to 0.70) (Table 2; Fig. 1). The calibration plot of the algorithm in the validation cohort showed calibration metrics with an intercept of 0.50 (95% CI 0.40 to 0.61) and a calibration slope of 0.90 (95% CI 0.74 to 1.04) (Fig. 2). The Brier score was lower than the respective null model Brier score (0.19 versus 0.20) indicating good overall performance of the SORG femoral neck fracture mortality algorithm. In the decision curve analysis, the SORG femoral neck fracture mortality algorithm has shown to provide a positive net benefit compared with a strategy of treating all patients or none as being at risk for 2-year mortality (Fig. 3). The model slightly underestimates the risk of 2-year mortality with predicted probabilities up to 60%, meaning that observed values may be higher than predicted.

### Available web-application

The externally validated algorithms were incorporated into a web-based application and deployed as open-access available tool for clinicians: https://sorg-apps.shinyapps.io/hipfracturemortality/

## Discussion

In this study, we externally validated the SORG femoral neck fracture mortality algorithm for predicting 90-day and 2-year mortality in femoral neck fracture patients aged 65 years or above in an independent intercontinental cohort derived from the Sheba Medical Center in Israel. We found that the SORG femoral neck fracture mortality algorithm, initially trained on a multicenter institutional North American cohort, performed acceptably on an institutional cohort from Israel. Calibration metrics, Brier score, and decision curve analyses suggest transferability of this algorithm to an independent intercontinental population, though poor discrimination warrants prospective evaluation to ensure feasibility and clinical corroboration in practice.

### Limitations

The results of this study should be viewed considering several limitations. First, the cohorts originated from different countries and continents, which may influence variations in (geriatric) treatment protocols and different education programs for orthopedic surgeons across countries. A previous study carried out a cross-cultural comparison of clinical outcomes following treatment in hip fracture patients in two different countries and found that although there were differences in protocols in the two countries that this did not influence the treatment outcomes practices [32]. In addition, implementation of geriatric-specific pathways are associated with lower costs and a shorter length of stay, but are not associated with influencing the mortality risk [33]. Therefore, we did not expect the differences from our cohort to influence treatment outcomes. Second, it must be emphasized that development and validation studies focus on developing and validating a clinical prediction model, rather than the explanation of this outcome (i.e., cause of mortality). Further, the generalizability of a prediction model is not ensured after a single external validation study and should be thoroughly evaluated in independent cohorts, if the cohort differs significantly in setting, patient demographics, and mortality incidence. Third, as the developed model include femoral neck fractures specific variables (i.e., displacement of the fracture using the Garden classification), the model could not be generalized to other locations (intertrochanteric/subtrochanteric). Future efforts can aim to use common data elements to translate location-specific models to a broader range of locations. In addition, the developmental [16] and current study focused on developing and externally validating a prediction model using variables that are available in the preoperative phase. Another perspective that may guide treatment decision-making is evaluating the individual treatment effect [34]. In prediction model research, the algorithm is used to predict an outcome (i.e., mortality) from given input variables (i.e., preoperative available variables). In causal research, statistical methods are used to evaluate the effect of an intervention or treatment (e.g., internal fixation or arthroplasty surgery) on the outcome (i.e., mortality). Subsequently, a model can investigate specific probabilities per treatment decision (e.g., internal fixation or arthroplasty). Lastly, a confounding factor for mortality estimation could be the presence of a do-not-resuscitate (DNR) order, precluding the use of cardiopulmonary resuscitation in a clinically unresponsive, pulseless patient. Surgical patients with DNR orders have higher mortality rates than those who do not have a DNR order [35]. Future efforts can evaluate end-of-life care directive data and their effect on the mortality estimation specific to the hip fracture population.

### Findings

We found that the SORG femoral neck fracture mortality algorithm, initially trained on a multicenter institutional North American cohort, performed acceptably on an institutional cohort from Israel. International validation studies with transparent reporting are an important step for moving prediction modeling from a single country to a coordinated global effort [36–38]. Though many promising clinical prediction models exist to predict mortality in hip fracture patients, the vast majority of them are awaiting external validation [17]. Our study highlights the importance of externally validating a well-developed algorithm in an independent intercontinental cohort. The current iteration of SORG performed with poor to acceptable discrimination on external validation in both 90-day and 2-year mortality. However, labeling systems for discrimination metrics are arbitrary [39]. High discriminatory ability is not directly sufficient to claim a positive potential effect of deploying a prediction model in clinical practice [39]. For clinical purposes, insights derived from a prediction model may go beyond model performance measures. The clinical context should determine what can be considered a reasonable performance looking at the decision threshold. Therefore, assessing the net benefit could serve as an initial assessment of clinical usefulness.

We interpreted the net benefit of the model with visualization in decision curve analyses. For 90-day mortality, the model was well calibrated in predicting patients up to 40% risk (Fig. 2), and the decision curve analysis suggests a threshold of 0.2 (Fig. 3). A threshold of 0.2 means that patients with a probability > 0.2 are classified as ‘positive’ and < 0.2 are classified as ‘negative’. For 2-year mortality, the model slightly underestimates the risk of 2-year mortality with predicted probabilities up to 60%, meaning that observed values may be higher than predicted (Fig. 2). The decision curve analysis shows to provide a net benefit suggested a threshold of 0.45, meaning that patients with a probability > 0.45 are classified as ‘positive’, and < 0.45 are classified as ‘negative’. These findings suggest that the model is being highly accurate in predicting patients at low risk of 90-day mortality, and low to moderate risk of 2-year mortality following femoral neck fracture surgery.

The Israeli cohort showed that a significant lower percentage of their population had comorbidities in comparison to the population included in the North American cohort. Previous studies have sought to explain the high rate of comorbidities in the USA, where nearly half (approximately 45% [40]) of all Americans suffer from at least one chronic disease and this difference can therefore be justified. The prediction model included three comorbidity features (i.e., CHF, hemiplegia and COPD) after feature selection in the development cohort, and although ML can work well at deriving associations and correlations, it cannot determine causation or assess whether those associations make physiologic sense.

Although this study shows promise in prognostication in patients sustaining a femoral neck fracture, further efforts are needed. The current study solely investigated the mortality risk estimation, future research can focus on investigating additional outcomes such as patient reported outcome measures (e.g., quality of life, symptoms of pain, and need for mobility-aid) or the risk of adverse events (e.g., reoperation). This will lead to a more patient-centered care approach and evaluating the individual patient’s needs. In addition, although patients with a femoral neck fracture are mostly treated surgically, a recent study showed that a shared decision-making process including nonoperative management for a proximal femoral fracture might be a viable option for frail institutionalized patients with limited life expectancy [41, 42].

## Conclusion

In conclusion, we have externally validated the SORG femoral neck fracture mortality algorithm, suggesting the transferability of this algorithm to an independent intercontinental population. We demonstrated the clinical utility, with the model being highly accurate in patients at low risk of mortality which may guide shared decision-making. Further studies are needed to evaluate this algorithm in a prospective setting and evaluate its feasibility and efficacy in practice.

## Supplementary Information

Below is the link to the electronic supplementary material.

Supplementary file1 (DOCX 88 KB)

## Data Availability

Data sharing not applicable to this article.
